# Radiology for surgeons: liver anatomy

**DOI:** 10.3389/fsurg.2026.1743615

**Published:** 2026-02-05

**Authors:** Kristina Marcinkevičiūtė, Ugnė Šilinskaitė, Raminta Lukšaitė-Lukštė, Mindaugas Kvietkauskas

**Affiliations:** 1 Faculty of Medicine, Vilnius University, Vilnius, Lithuania; 2 Department of Radiology, Nuclear Medicine and Medical Physics, Institute of Biomedical Sciences, Faculty of Medicine, Vilnius University, Vilnius, Lithuania; 3 Clinic of Gastroenterology, Nephrourology, and Surgery, Institute of Clinical Medicine, Faculty of Medicine, Vilnius University, Vilnius, Lithuania; 4 Laboratory of Experimental Surgery and Oncology, Translational Health Research Institute, Faculty of Medicine, Vilnius University, Vilnius, Lithuania

**Keywords:** anatomy, hepatobiliary surgery, liver, radiology, variations

## Abstract

The liver encompasses the portal vein, hepatic arteries, hepatic veins, and biliary ducts, all of which display considerable anatomical variation with clinical and surgical relevance. This study aimed to summarize this anatomical variability and its impact on decision-making in surgery. A review of studies and case reports was conducted, focusing on documented anatomical variations. The most commonly observed variations were highlighted using imaging findings. The study found that the anatomy of the liver’s vascular and biliary systems differs in up to 50% of cases; therefore, in hepato-pancreato-biliary surgery, the surgeon’s radiology knowledge and pre-operative planning with a specialized radiologist are particularly important. Pre-operative collaboration with radiologists would allow for more accurate mapping of the vascular and biliary anatomy. This review highlights the necessity of precise knowledge of liver anatomy and the importance of interdisciplinary collaboration between radiologists, surgeons, and oncologists to improve patient outcomes.

## Introduction

Liver anatomy is complex and characterized by its lobes, segment classification, and varying vascular and biliary systems. Hepatic resection necessitates an adequate future liver remnant with preserved inflow, outflow, and bile drainage to ensure proper liver function post-surgery (1). Therefore, to avoid complications during surgery, it is crucial for surgeons to have a thorough understanding of liver anatomy, especially for major hepatobiliary surgery, including transplantations. Radiological insights provide a comprehensive understanding and different perspectives of liver anatomy, helping surgeons plan each step of the surgery and consequently improving patients' outcomes. Thus, advanced radiology knowledge is crucial for surgeons, helping them to collaborate effectively with radiologists and achieve the best postoperative results. A precise understanding of anatomical relationships can significantly affect tumor management by delineating surgical limitations and influencing the choice between chemotherapy or radiotherapy over surgical intervention. This article covers essential liver radiology concepts for surgeons.

## Methods

This review summarizes the anatomy of the liver, including its vascular and biliary systems, and discusses the most appropriate imaging techniques. A literature search was conducted using the PubMed database to identify relevant articles. In total, 37 studies were included in the review. No restrictions were applied regarding the year of publication. However, only human studies were included in the review. Inclusion was limited to articles published in English or Lithuanian with full-text availability.

### Liver anatomical and functional divisions

The liver can be divided into four anatomical lobes, namely, the larger right, smaller left, quadrate, and caudate lobes (2). The falciform ligament divides the diaphragmatic surface into the right and left lobes (3). On the visceral surface, all four lobes can be seen. The quadrate lobe is separated by the *ligamentum teres* (on the left), the gallbladder fossa (on the right), and the porta hepatis (above). The caudate lobe is situated among the *ligamentum venosum* (on the left), the *vena cava* groove (on the right), and the porta hepatis (below) (4).

Unlike the anatomical divisions, the liver is divided into two functional parts (4): the left lobe and the right lobe. These parts are separated by the middle hepatic vein, which is called Cantlie's line. Cantlie's line is an imaginary line between the gallbladder fossa and the left side of the inferior vena cava posteriorly.

**Figure 1:**
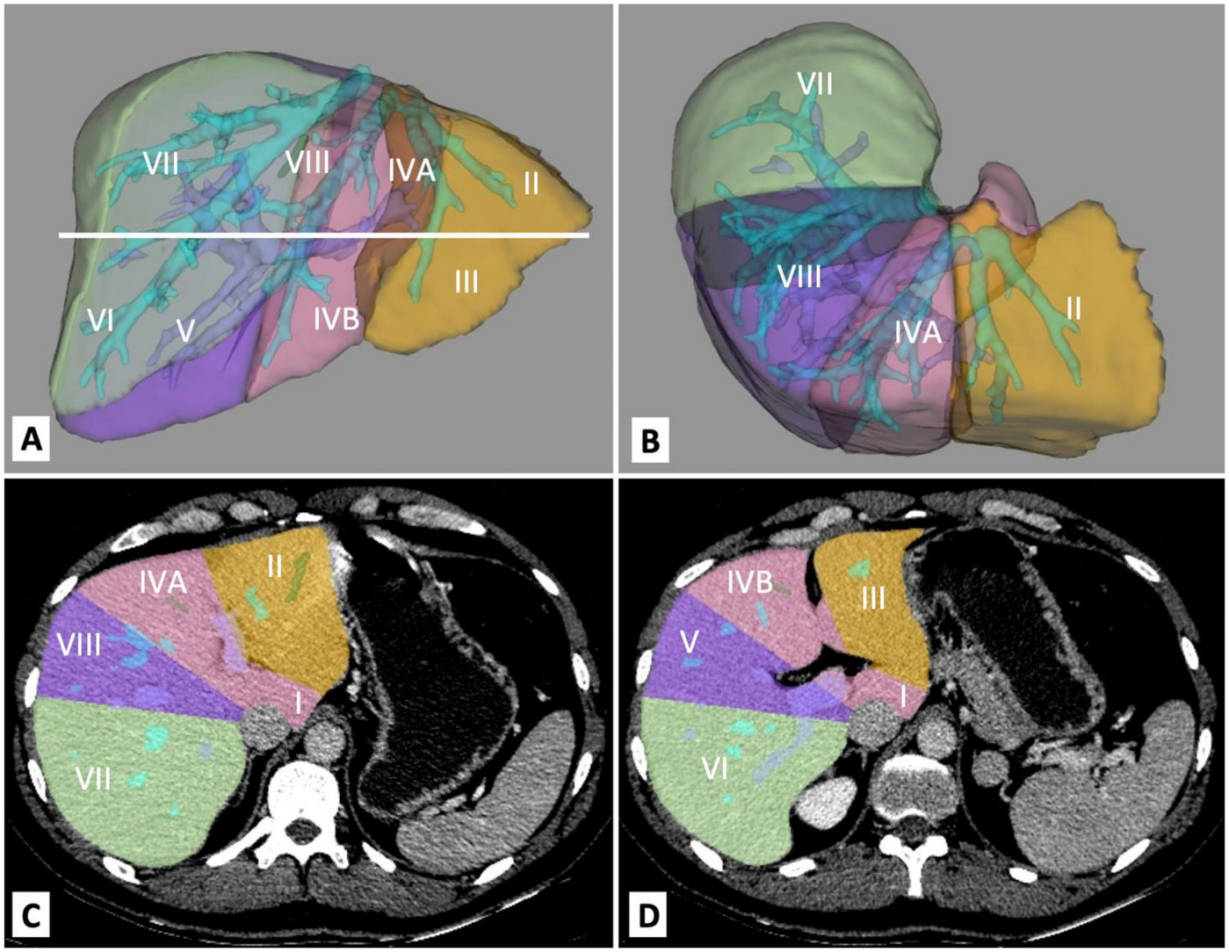
3D computed tomography reconstruction of the liver with segmental anatomy: **(A)** anterior view of the liver; **(B)** superior view of the liver. Liver segments in computed tomography: **(C)** superior cross-section of the liver; **(D)** inferior cross-section of the liver.

The Couinaud classification has been used since 1957 (5). It divides the liver into eight independent segments. Each segment has a hepatic arterial branch, a portal branch, and a bile duct with a separate hepatic venous branch (3). The numbering of the segments is done clockwise (Figures 1A,B). Segments II and III are in the left anatomical lobe. Together, they are referred to as the left lateral segment of the liver or the topographic left lobe. Segment IV (a and b) is situated between the falciform ligament and Cantlie's line. Segments II, III, and IV form the functional left part of the liver. The functional right side of the liver comprises segments V–VIII (3). V and VIII are anterior segments, and VI and VII are posterior segments. The caudate lobe is the first segment and can hardly be seen from the anterior side.

Computed tomography (CT) and magnetic resonance imaging (MRI) are used for volumetric or morphological analysis (6). While CT is more commonly used due to wider accessibility in clinical practice (Figures 1C,D), MRI minimizes the risk of nephrotoxicity and reduces radiation exposure(7). Ultrasound (US) should not be used to measure segments or volume due to its low accuracy when measuring liver diameter (8). Therefore, CT remains the gold standard for measuring segments and volume.

### Liver arteries

#### Anatomy of liver arteries

In a standard case, arterial blood to the liver is provided by the aorta, which supplies blood through the celiac trunk and the common hepatic artery, which forms two branches: the proper hepatic artery (PHA) and the gastroduodenal artery. This split usually occurs at the omental foramen. The PHA bifurcates into the right (RHA) and left hepatic (LHA) arteries. The RHA is routed anteriorly to the portal vein (PV) and on the left side, posteriorly to the common bile duct (CBD). The RHA culminates in the anterior segmental artery, which supplies segments V and VIII, and the posterior segmental artery, which supplies segments VI and VII. In addition, the RHA usually supplies the cystic artery, which in turn supplies the gallbladder. The LHA runs vertically toward the umbilical fissure, and its segmental arterial branches supply segments I–III. A middle hepatic artery usually branches off the LHA and runs toward the right side of the umbilical fissure and, in one-third of cases, supplies the IV segment (9). Nevertheless, in the majority of cases, the IV segment is supplied by both the RHA and the LHA (9).

#### Variations of liver arteries

**Figure 2:**
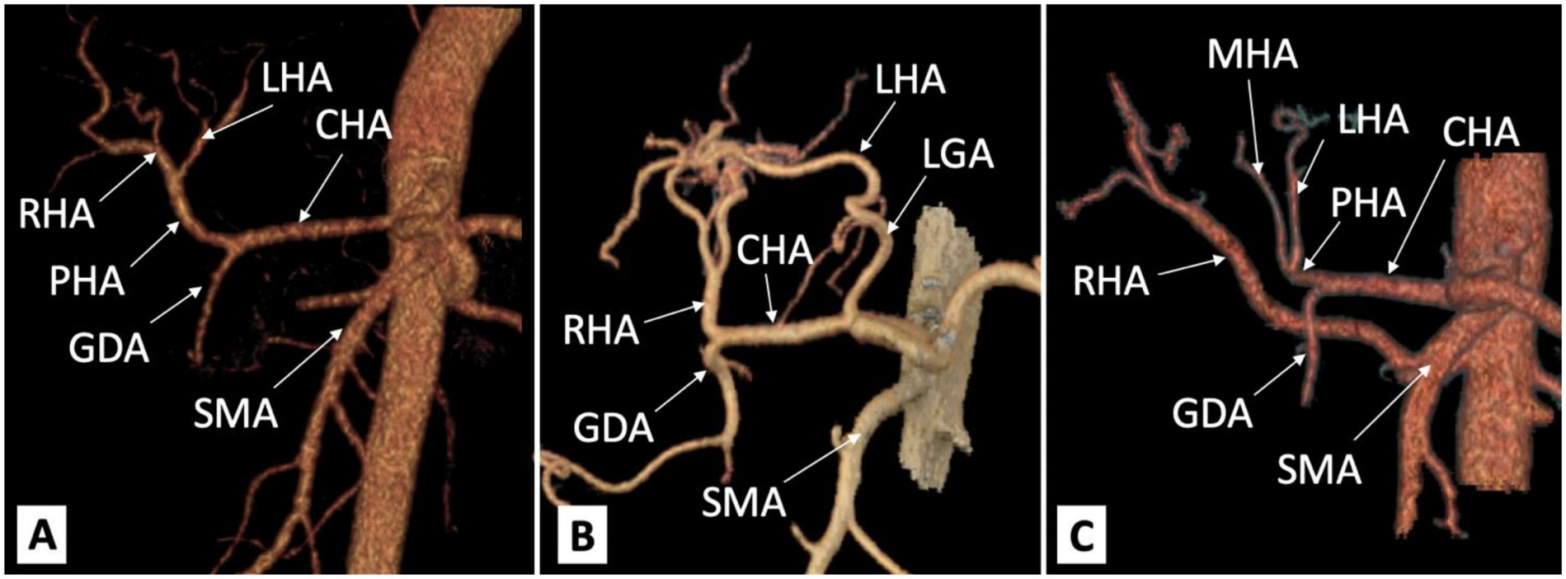
Common hepatic arterial variants on 3D computed tomography reconstruction: **(A)** Michel's type I (normal liver artery anatomy); **(B)** Michel's type II (LHA from LGA); **(C)** Michel's type III (RHA from SMA). CHA, common hepatic artery; GDA, gastroduodenal artery; LGA, left gastric artery; LHA, left hepatic artery; PHA, proper hepatic artery; RHA, right hepatic artery; SMA, superior mesenteric artery.

The prevalence of liver artery variations ranges from 20% to 50% of cases in different studies (10–12). The prevalence of aberrant arteries differs between studies (12, 13). Approximately 30% of patients have aberrant hepatic arteries, with similar prevalence rates for aberrant RHAs (15%) and aberrant LHAs (16%), and approximately 5% of the patients have both aberrant arteries (13). However, a previous study reported that an aberrant or accessory left hepatic artery arising from the left gastric artery was present in 3.0% of patients, while an aberrant or accessory right hepatic artery originating from the superior mesenteric artery was observed in 11.9% of cases (12). A combination of anomalies in both the LHA and RHA was observed in only 1.4% of the cases (12). One of the most popular classification systems for these variations is Michel’s classification. This is described in Table 1. The first three types are the most prevalent and are shown in Figure 2.

**Table 1:** Variation in liver artery anatomy and its prevalence according to Michel’s classification system (10, 11).

Type	Description	Prevalence (%)
I	Normal anatomy	50.0–80.0
II	LHA from the LGA	2.5–16.3
III	RHA from the SMA	6.0–15.5
IV	LHA from the LGA and RHA from the SMA	1.0–7.4
V	Acc. LHA from the LGA	0.6–3.2
VI	Acc. RHA from the SMA	0.4–1.6
VII	Acc. LHA from the LGA and acc. RHA from the SMA	∼0.2
VIII	Acc. LHA from the LGA and RHA from the SMA	∼0.35
IX	CHA from the SMA	∼1.2
X	CHA from the LGA	∼0.04

CHA, common hepatic artery; LGA, left gastric artery; LHA, left hepatic artery; RHA, right hepatic artery; SMA, superior mesenteric artery.

Hiatt’s classification system proposed a revised categorization of hepatic arterial anatomy, categorizing variations into six distinct types (14). In addition to this classification system, the study reported the frequency of each variant based on a cohort of 1,000 donors. Type 1 (*n* = 757) represents the typical arterial anatomy, in which the common hepatic artery arises from the celiac trunk and gives rise to the gastroduodenal artery and the proper hepatic artery. The proper hepatic artery then divides into the right and left hepatic branches. Types 2–6 represent less frequently encountered arterial variations (Table 2).

**Table 2:** Hiatt’s classification system for hepatic arterial anatomy.

Type	Description	Number of cases (*n* = 1,000)
Type 1	Typical arterial anatomy	757
Type 2	A replaced or accessory left hepatic artery arises from the left gastric artery	97
Type 3	A replaced or accessory right hepatic artery originates from the superior mesenteric artery	106
Type 4	In this double-replaced pattern, the right hepatic artery arises from the superior mesenteric artery, and the left hepatic artery is a branch of the left gastric artery	23
Type 5	The entire common hepatic artery originates as a branch of the superior mesenteric artery	15
Type 6	The common hepatic artery originates directly from the aorta	2

#### Imaging techniques for liver arteries

CT angiography is the preferred method for preoperative hepatic artery imaging because of its high spatial resolution, fast acquisition time, and low susceptibility to motion (15). In comparison, contrast-enhanced magnetic resonance (MR) angiography is an excellent quality imaging tool; however, prolonged breath holding is needed for better-quality images (16). Doppler US is used to evaluate liver transplants for arterial occlusion (17), which is identified by an absence of color.

### Portal veins

#### Anatomy and variations of the portal vein

Approximately 80% of the blood (3) flowing to the liver comes from the portal vein, which is usually formed by the fusion of the splenic and superior mesenteric veins. The inferior mesenteric vein most commonly drains into the splenic vein (3), but it can also drain into the superior mesenteric vein or the splenomesenteric confluence (18). The coronary (left gastric) vein, cystic vein, and tributaries of the right gastric and pancreaticoduodenal veins drain directly into the portal vein (19). There are rarer types of variations in portal venous anatomy (described in the literature as being prevalent in up to 5.2% of cases). These variations involve different patterns of formation and drainage of the PV, superior mesenteric vein (SMV), inferior mesenteric vein (IMV), accessory mesenteric vein (AccMV), left gastric vein (LGV), and splenic vein (SV). These variants include alternative PV formation (e.g., from two SMVs or with IMV contribution), variable tributary drainage of the IMV, AccMV, and LGV, duplication or absence of the IMV, and uncommon venous configurations (20).

**Figure 3:**
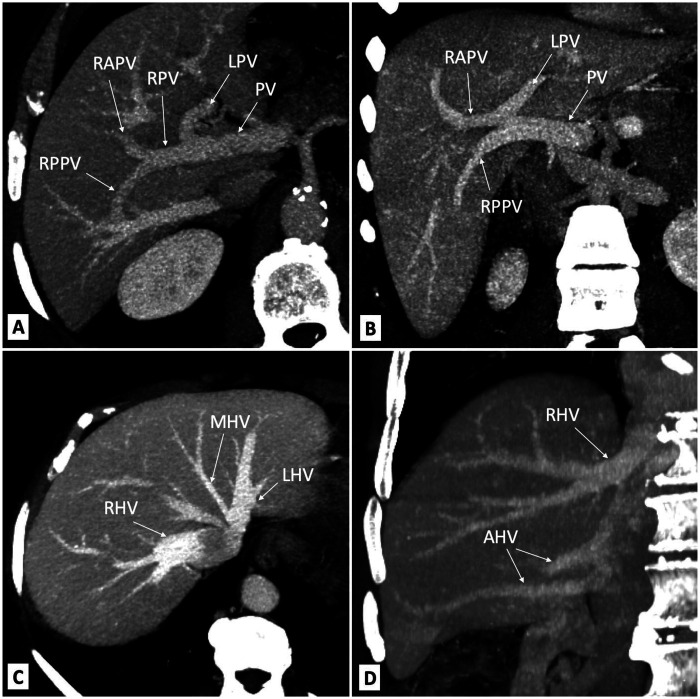
Portal vein trifurcation in liver computed tomography: **(A)** Nakamura's type A and Cheng's type I (bifurcation of the main PV into the LPV and RPV); **(B)** Nakamura's type B and Cheng's type II (trifurcation of the main PV into the LPV, RAPV, and RPPV; the common RPV is missing). Hepatic veins on computed tomography images: **(C)** main trunks of hepatic veins; **(D)** accessory hepatic veins. AHV, accessory hepatic vein; LHV, left hepatic vein; LPV, left portal vein; MHV, middle hepatic vein; PV, portal vein; RAPV, right anterior portal vein; RHV, right hepatic vein; RPPV, right posterior portal vein; RPV, right portal vein.

Further branching of the portal vein has been classified by Nakamura et al. (21) and Cheng et al. (22) (Table 3). Typical portal vein branching occurs in up to 80% of the population (18, 23), and the most common variation is portal vein trifurcation (Figures 3A,B). The portal vein divides the liver into upper and lower segments (3). The portal vein enters the liver on the posterior side and divides into two branches, the right and left portal veins, near the liver hilum. The left portal vein supplies blood to the caudate lobe and segments II, III, and IVa/b. The right portal vein divides into posterior and anterior branches (19). The posterior branch of the right portal vein supplies blood to segments VI and VII, while the anterior branch of the right portal vein supplies blood to segments V and VIII (24).

**Table 3:** Portal vein classification according to Nakamura et al. (21) and Cheng et al. (22).

Type	Classification		Definition
	Nakamura et al.	Cheng et al.	
	A	I	Normal anatomy: bifurcation of the main PV into the LPV and RPV
	B	II	Most common variation—a trifurcation of the main PV into the LPV, RAPV, and RPPV. The common RPV is missing
	C	III	The RPPV arises separately from the main PV, followed by extraparenchymal bifurcation into the RAPV and LPV intraparenchymal
	D	IV	The RPPV arises separately from the main PV, followed by intraparenchymal branching of the RAPV
	E	IV	Separate PV branches for liver segments IV, V, and VIII

LPV, left portal vein; PV, portal vein; RAPV, right anterior portal vein; RPPV, right posterior portal vein; RPV, right portal vein.

#### Imaging techniques for portal veins

Doppler US is useful for evaluating the portal venous system as US itself is non-invasive, inexpensive, portable, generally well-tolerated by patients, and presents no ionizing radiation (16, 25–27). Moreover, it is widely available and provides insight into venous flow (not just anatomical images) (25). However, the quality of the view depends heavily on the patient's cooperation and biotype (26), along with the operator’s ability (16). Therefore, in some cases, other imaging techniques are required after Doppler US (26). In the majority of facilities, CT is the preferred imaging method for the portal venous system due to its ability to quickly provide high-resolution images and create three-dimensional reconstructions and high-quality multiplanar reformations (26). MRI can also be used. In comparison to CT, MRI's advantages are the absence of intravenous contrast and radiation during the procedure (26). However, it is more expensive, takes longer, and is less accessible. It also has poorer spatial and temporal resolution and is more susceptible to artifacts (27).

### Hepatic veins

#### Anatomy and variations of hepatic veins

The liver's blood outflow is carried through the right, middle, and left hepatic veins (3) (Figure 3C). The right hepatic vein typically drains directly into the inferior vena cava (IVC) (19). The left and middle hepatic veins may drain directly into the IVC; however, more commonly, they merge and form a short trunk before entering the IVC (19). This common trunk anatomy is found in approximately 70%–81% of cases (27–29). An accessory inferior right hepatic vein is also present in up to 48% of the population (29, 30) (Figure 3D).

#### Imaging techniques for hepatic veins

Hepatic vein radiology is less extensively researched compared to that of hepatic arteries and portal veins. The US is the first-line imaging tool due to its accessibility and cost-effectiveness. Additionally, it provides real-time imaging; however, the results depend heavily on the operator's experience. Moreover, some cardiological conditions, such as sinus bradycardia or sinus tachycardia, can influence the results of Doppler US (31). CT provides a detailed anatomical view and assessment of the surrounding structures (16, 32). MRI additionally provides information on soft tissues, liver lesions, and their stiffness (32).

### Biliary tree

#### Anatomy of the biliary tree

Bile is produced in the liver parenchyma and is delivered by the biliary tree to the gallbladder and the duodenum. The biliary tree consists of the intrahepatic and extrahepatic bile ducts. The right anterior sectoral duct (RASD), which delivers bile from liver segments V and VIII, together with the right posterior sectoral duct (RPSD), which delivers bile from liver segments VI and VII, forms the right hepatic duct (RHD). The RHD merges with the left hepatic duct (LHD) to form the common hepatic duct (CHD). A small duct from the I (caudate) lobe drains bile into this junction (33, 34).

#### Variations of the biliary tree

Variations in the anatomy of the biliary tree are common and may only be detected during surgery or intervention. Extrahepatic variants increase the technical difficulty of procedures such as cholecystectomy and increase the risk of an iatrogenic bile duct injury, leading to bile leaks postoperatively.

**Figure 4:**
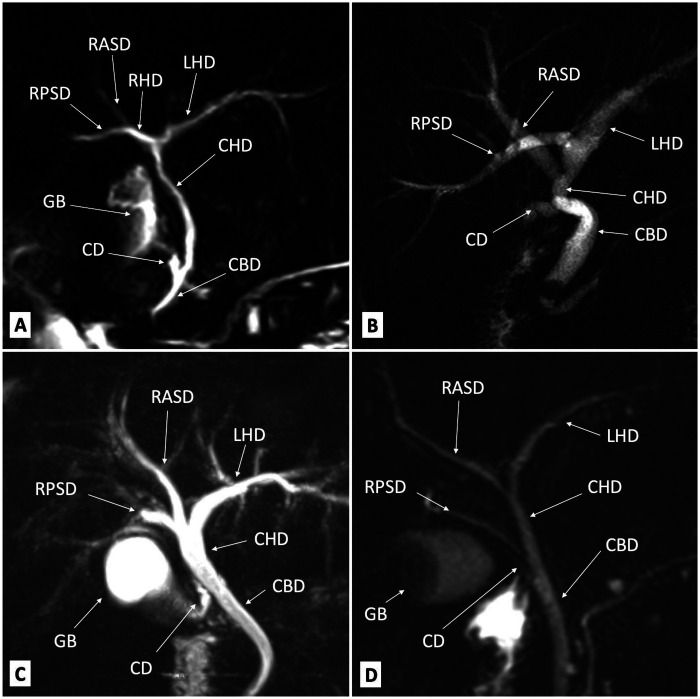
Magnetic resonance cholangiography images of the bile ducts: **(A)** normal anatomy; **(B)** the RPSD drains directly into the LHD without connection with the RASD; **(C)** RPSD, RASD, and LHD triple confluence; **(D)** low inflow from the RPSD directly into the CHD above the CD. CD, cystic duct; CBD, common bile duct; CHD, common hepatic duct; GB, gallbladder; LHD, left hepatic duct; RASD, right anterior sectoral duct; RHD, right hepatic duct; RPSD, right posterior sectoral duct.

The cystic duct from the gallbladder passes into the right side of the CHD and, together with the CHD, forms the CBD. The CBD passes through the free edge of the lesser omentum and runs posteriorly to the duodenum and anteriorly to the inferior vena cava (31). The CBD merges with the main pancreatic duct to form the ampulla of Vater, which drains into the major duodenal papillae in the D2 segment of the duodenum. Normal biliary tree anatomy is present in up to 60% of the population (35, 36) (Figure 4A). The most common intrahepatic biliary tree variation, i.e., when the RPSD drains directly into the LHD without merging with the RASD, is reported in 13%–19% of cases (35) (Figure 4B). In 11%–12% of the population, the RPSD drains into the anterior, instead of the posterior, side of the RASD or RPSD, together with the RASD and LHD, forming the CHD through a triple confluence (Figure 4C). Other intrahepatic variations are rarely presented in the literature (Figure 4D). The three most common extrahepatic biliary tree variations are cystic duct medial insertion from the left side of the CHD (15% frequency), cystic duct insertion into the distal third of the CHD (10%), or a parallel path to the CHD of 2 cm or more (10%) (37).

#### Imaging techniques for the biliary tree

CT cholangiography with contrast was previously used to image the biliary tree, but due to adverse reactions to the contrast agents and lower performance, it has been replaced by MR cholangiography (MRC) (15, 38). 2D MRC images can be taken during a single breath hold, but they can be affected if there are superimposed fluid-filled structures (e.g., the stomach or duodenum). This can lead to an incomplete evaluation of the biliary tree. 3D MRC provides better visualization of smaller-diameter intrahepatic structures and can create a 3D reconstruction; however, it is usually longer (13). Studies have shown that when 2D and 3D MRC are combined, their accuracy when depicting biliary anatomy reaches 84.6%–90.4% (39).

From a clinical standpoint, awareness of anatomical variations is crucial across multiple medical specialties. For radiologists, this knowledge enhances accurate imaging interpretation and pre-operative mapping, particularly by allowing them to distinguish normal variants from pathological findings. For surgeons, it can inform the surgical strategy and reduce the risk of iatrogenic injury during resection or transplantation. For oncologists, these anatomical considerations are important for precise tumor localization and regional treatment planning, enabling more accurate staging and optimized therapeutic decision-making.

### Summary

Variations in liver vascular and biliary anatomy are relatively common; therefore, it is crucial for surgeons to be knowledgeable about these variations in order to minimize complications. This article provides a detailed summary of typical anatomy and variations of liver structures and their representation in imaging tests. However, no single imaging modality is the best universal technique for assessing hepatic arteries and veins, portal veins, and biliary structures. Each imaging method has drawbacks, requiring each patient's situation to be assessed individually. Moreover, a radiological assessment of liver structures provides operating surgeons and treating oncologists with additional anatomical insights. Thus, it can significantly impact surgical approaches, the possibility of radiotherapy and chemotherapy, and, consequently, patient outcomes.
